# In Vivo Local Administration of Para-Amino-Bebblistatin to the Injured Spinal Cord Fails to Improve the NaChBac-Expressing DRGs Transplantation

**DOI:** 10.3390/ijms262110479

**Published:** 2025-10-28

**Authors:** Sonia Hingorani, Guillem Paniagua Soriano, Carlos Sánchez Huertas, Victoria Moreno Manzano

**Affiliations:** 1 Neuronal and Tissue Regeneration Laboratory, Centro de Investigación Príncipe Felipe (CIPF), 03202 Valencia, Spain; gpaniagua@cipf.es; 2 Development and Assembly of Bilateral Neural Circuits Laboratory, Instituto de Neurociencias, Consejo Superior de Investigaciones Científicas (CSIC), Universidad Miguel Hernández, 03550 Alicante, Spain; chuertas@umh.es

**Keywords:** cytoskeleton, sensory neuronal cultures, spinal cord injury, blebbistatin, para-amino-blebbistatin, epothilone B, NaChBac

## Abstract

Spinal cord injury (SCI) is a devastating, debilitating, and life-altering condition that lacks a cure or effective treatment as of today. An altered excitation/inhibition ratio after an injury, with an increase in inhibitory input, limits motor and sensory function. Together with the limited endogenous regeneration capacity of the affected neuronal circuits, this results in further loss of function. Hingorani and collaborators recently reported that transplantation of dissociated sensory neurons from neonatal dorsal root ganglia (DRGs) expressing the bacterial sodium channel NaChBac significantly improved locomotion in a severe SCI by increasing the excitatory neuronal input at the injury site. Here, we additionally target the potential axonal regeneration of endogenous and transplanted cells, using cytoskeleton-modulating drugs to enhance axonal length. We employ, alone or in combination, blebbistatin and epothilone B, tested in vitro. We found that individually, each compound significantly induced the sensory neurons’ axonal elongation; however, their combination completely abolished it. Interestingly, a combinatory treatment including the modification of DRGs to express the NaChBac sodium channel and the treatment with blebbistatin increased the axonal elongation in vitro. Nevertheless, when applied in vivo in a model of SCI, local and single para-amino-blebbistatin (a stable analogue of blebbistatin) administration and the transplanted NaChBac expressing sensory neurons limit the functional recovery enabled by neuronal transplantation alone. Thus, despite the beneficial outputs of isolated neuronal cultures that allow selection of in vivo combinatory strategies, the multifaced characteristics of CNS injuries limit the potential success of single and local treatment administration, demanding extended and sustained treatments.

## 1. Introduction

During development, central nervous system (CNS) axons display a high intrinsic ability to grow and innervate their target. However, as they mature, the CNS neurons lose this capacity, including after an injury. This limitation is attributed to several factors such as the lack of appropriate regenerative machinery governed by growth-associated genes, which are suppressed after injury [1]. On the other hand, the peripheral nervous system (PNS) retains its intrinsic capacity to regenerate until adulthood [2]. For the above-mentioned, the exploitation of increased regenerative potential and support of the PNS has already been described in early spinal cord injury (SCI) studies as a therapeutic strategy, initially established by Aguayo and colleagues employing peripheral nerve grafts [3], and lastly in the recent report by Hingorani et al. [4]. The role of the peripheral nervous system as a potentiator for CNS repair is further highlighted by the “conditioning lesion paradigm”, a well-studied phenomenon where a previous insult to the peripheral axon of DRG leads to a switch in the regenerative capacity of the central axon, mediated by the upregulation of several RAGs after increased levels of intracellular Ca++ and cyclic adenosine monophosphate (cAMP) at the DRGs, even after SCI [5]. Earlier studies from Silver’s lab concluded that minimally damaging transplants by microinjections of postnatal or adult dissociated DRGs, rostral to the injured brain, project long axons and robustly integrate, forming multiple collaterals into the white matter of the host adult rat brain [6]. In the adult spinal cord, micro-transplanted dissociated adult DRG neurons, when transplanted distal to the primary trauma, were able to survive and extend up to 4–5 mm in rats; however, they were unable to invade the inhibitory scar, thus limiting their capacity to cross over the injury and contribute to denervated circuits caudal to the lesion in this case [7]. Therefore, we proposed to improve the regenerative capacities of the transplanted DRGs by increasing their intrinsic neuronal activity through the ectopic expression of NaChBac, a sodium-gated channel first identified in 2001 [8], which supports higher neuronal circuit integration [9] and prevents activity-dependent death [10]. We found that NaChBac-expressing DRG transplantation significantly increases locomotor recovery when compared to non-modified DRG transplanted and non-transplanted animals by increasing the number of excitatory VGLUT2 inputs within the relay zone and caudal to the injury. However, the DRG transplanted cells failed to cross through the injury site, limiting the opportunities to form new neuronal relays within the host neurons [4]. Therefore, we further hypothesized that additional treatment involving cytoskeleton-modulating drugs to improve the axonal length would also contribute to overcoming the inhibitory stimulus such as the presence of inhibitory chondroitin sulphate proteoglycans (CSPGs) within the generated scar matrix [11], of both transplanted cells and local neurons, along with previously described DRG transplants, to improve connectivity and transplant survival.

Several pharmacological agents have enabled axons to overcome the inhibitory effect of the CSPGs. Some such are cytoskeleton modulating drugs that modulate the growth cone to overcome axonal retraction and promote axonal regeneration, such as blebbistatin and epothilone B. Blebbistatin, a pharmacological inhibitor of Non-muscle myosin II (NMMII), a downstream effector of the Rho/ROCK pathway and responsible for retrograde actin flow in the growth cone, effectively enhanced axonal growth in presence of CSPGs and myelin in vitro [12]. Epothilone B, a microtubule-stabilizing agent that can cross the blood–brain barrier (BBB), improved axonal regeneration after SCI in rats [13]. More recently, we have also shown that epothilone B alone reduced fibrotic scarring, as well as decreased the infiltration of cells into the injury area. Additionally, it significantly improved serotonergic regeneration and increased vesicular glutamate transporter 1 (VGLUT1) excitatory input into the central pattern generator, thereby improving functional recovery. In combination with rehabilitation, all of these parameters were further enhanced [14]. The evaluation of the involved mechanisms can be performed in neurons in culture, serving as essential models to shed light on the specific molecular cascades underlying injury repair and identify therapeutic targets. One of the greatest pathological features of SCI is direct damage to axonal tracts. Hence, axotomies and neurite outgrowth assays are a good in vitro model and their use as such is widespread in SCI research. In fact, several SCI treatments focus on directly aiming for improving axonal regeneration [15]. Here, we first hypothesize that blebbistatin and epothilone B, individually reported to induce axonal elongation through different mechanisms of action [12,16], may have a synergistic effect on neurite length. Next, we studied the effect of the combination with DRG transplantation in an in vivo SCI model. In order to maximize the synergy of the treatment and effectiveness of the SCI recovery, we also test it in combination with the modification of the sensory neurons to express the bacterial sodium channel NaChBac.

## 2. Results

### 2.1. Blebbistatin or Epothilone B Alone Rescues the Neurite Length of Neurons Plated over CSPGs, Whereas a Combination Reverts This Effect

**Figure 1:**
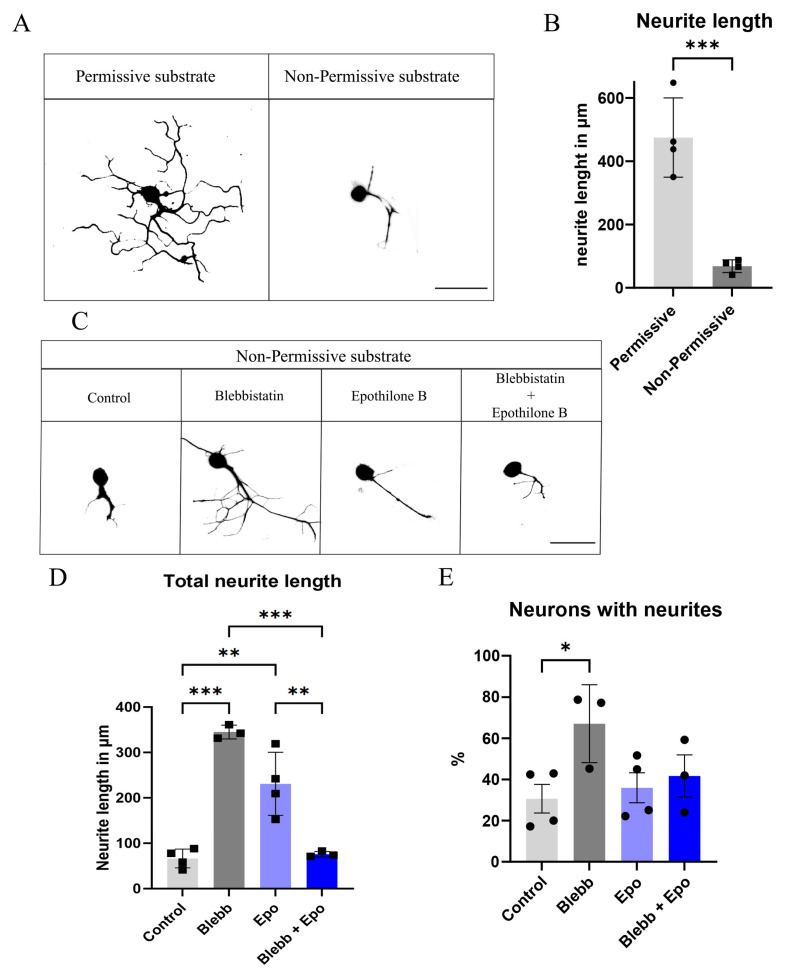
Blebbistatin or epothilone B increases the neurite length of neurons, while their combination is detrimental: (**A**) Images illustrating the neurite length of neurons plated over Permissive and Non-Permissive substrates. (**B**) Graphical quantification of neurite length, *** *p* = 0.0007, Student’s *t*-test. (**C**) Images illustrating the neurite length of neurons plated over Non-Permissive substrates in the different conditions. (**D**) Quantification of DRG neurons’ neurite length over Non-Permissive substrate in the different conditions. *** *p* < 0.0002, ** *p* < 0.003; Two-way ANOVA followed by Tukey’s test. (**E**) Percentage of DRG neurons with neurites when plated over Non-Permissive substrate in the different conditions. * *p* = 0.04, Two-way ANOVA followed by Tukey’s test. All data are represented as Mean ± SD, n = 3–4.

We first verified that the Non-Permissive CSPGs did indeed create an inhibitory environment and reduced neurite length and outgrowth, similar to that found in the injury milieu. We generated “Permissive” (laminin and 2 µg/mL bovine serum albumin (BSA)) and “Non-Permissive” (laminin and 2 µg/mL CSPGs) coatings and found a significant decrease in DRG’s neurite length in the Non-Permissive condition (Figure 1A,B). To observe a possible synergistic effect with the drugs, we analyzed the effect of blebbistatin (Blebb) and epothilone B (Epo), both separately and together. Our studies showed a significant increase in neurite length and percentage of DRG neurons with neurites treated with blebbistatin, whereas the combination of both drugs seemed to reverse this effect. Indeed, we found a significant decrease in the neurite length of DRGs treated with a combination of blebbistatin and epothilone B (Figure 1C–E).

### 2.2. Growth Cone Area Increases with the Presence of CSPGs, Blebbistatin Reverts Back to Normal Conditions, Whereas the Combination with Epothilone B Fails to Do So

**Figure 2:**
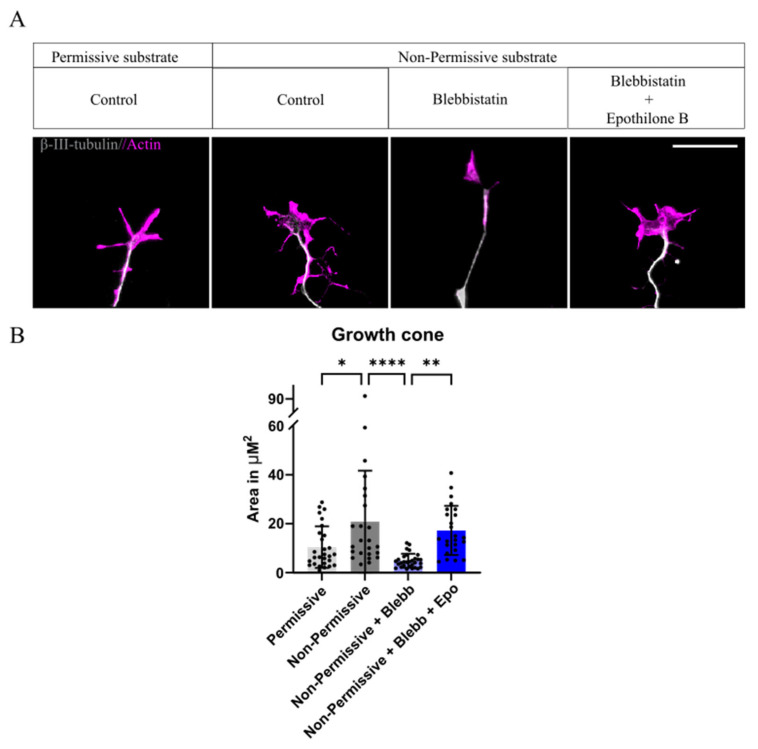
Blebbistatin decreases growth cone area, whereas CSPGs and combinatory treatment increase its area: (**A**) Images illustrating the growth cone structure of neurons plated over Permissive and Non-Permissive substrates in the different conditions. (**B**) Differences in growth cone area in the different conditions, * *p* = 0.01, ** *p* = 0.001, **** *p* < 0.0001, One-way ANOVA followed by Tukey’s test, n = 4. All data are represented as Mean ± SD.

Since we observed that the combination of both drugs resulted in a decrease in axonal length, we decided to acquire insight into the possible reason behind this effect. It has been previously reported that, when plated over CSPGs, an increase in the growth cone area, namely, a “splayed” growth cone structure, leads to shorter axonal lengths. Treatment with blebbistatin returns this structure to a thinner, tightly bundled growth cone, which facilitates axonal outgrowth [16]. We analyzed the growth cone structure, defined as the actin-positive area of the growth cone, when treated with either blebbistatin, or the combination with epothilone B. We found that Non-Permissive coating significantly increased the growth cone area of DRGs, leading to a “splayed structure” (Figure 2A). As expected, treatment with blebbistatin reverts this effect; however, its combination with epothilone B disrupts this and restores the splayed growth cone structure (Figure 2A,B), explaining the combinatory treatment failure to increase axonal length.

Our studies suggest that the combination of epothilone B and blebbistatin presents an undesired effect rather than the expected synergistic one and reverts to the axonal length similar to that found in Non-Permissive conditions without any treatment. Therefore, we discarded the combination strategy and focused on blebbistatin alone.

### 2.3. Blebbistatin Increases Neurite Length in Neurons Expressing NaChBac Under Non-Permissive Substrate

**Figure 3:**
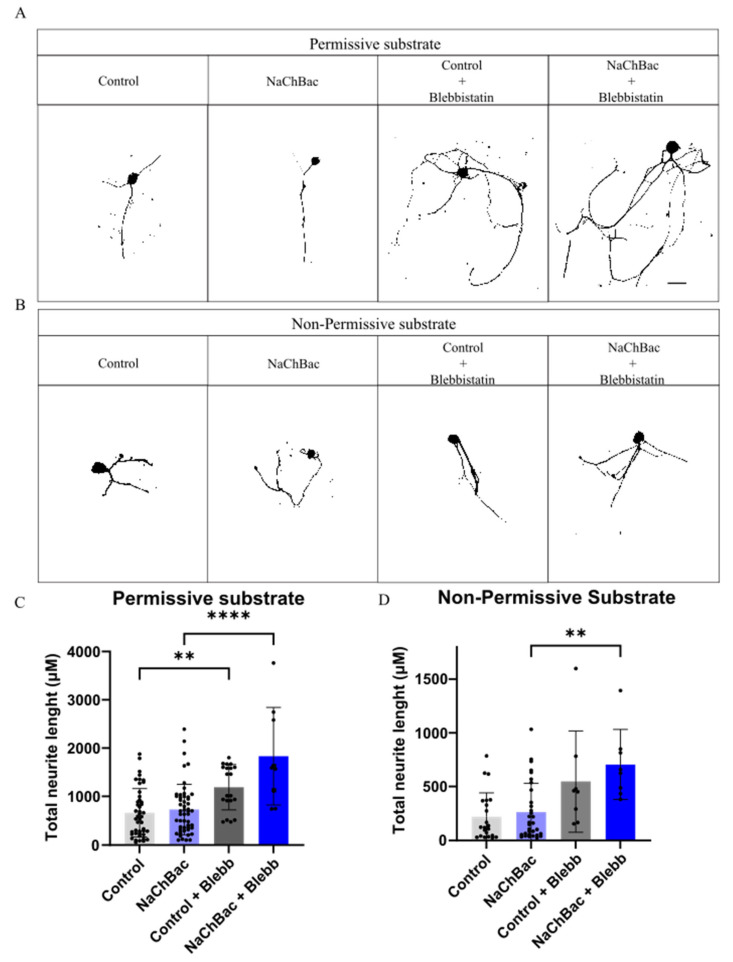
Effect of combinatory treatment of NaChBac and blebbistatin over dissociated DRG neurons: (**A**) Images illustrating the neurite length of DRG neurons expressing Control or NaChBac vectors re-plated over Permissive and (**B**) Non-Permissive substrates. (**C**) Quantification of the neurite length of these DRG neurons plated over Permissive substrate (** *p* = 0.0029, **** *p* < 0.0001) or (**D**) over Non-Permissive substrate (** *p* = 0.0015). One-way ANOVA followed by Tukey’s test. n = 3. Data presented as mean ± SD.

We have recently reported in a severe mouse SCI model that the acute transplantation of dissociated DRGs modified to express the bacterial sodium channel NaChBac is an effective treatment for improving motor locomotion recovery [4]. Thus, we wanted to test whether we could boost recovery by combining both strategies before moving to the in vivo model. We first analyzed its effect on neurite length in vitro and found that NaChBac alone does not improve neurite length, in either substrate type. Nevertheless, treatment with blebbistatin significantly increases neurite length of NaChBac expressing neurons both under Permissive (Figure 3A,C) and Non-Permissive coatings (Figure 3B,D). These results validated the idea of combining NaChBac with blebbistatin in the in vivo model for an increased motor recovery.

### 2.4. Administration of Para-Amino-Blebbistatin Does Not Alter Functional Locomotion After SCI

**Figure 4:**
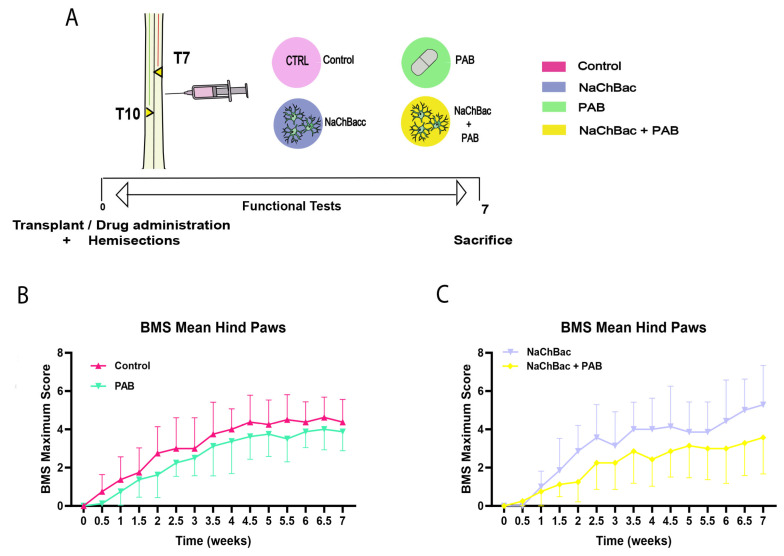
Functional recovery evaluation of combinatory therapy: (**A**) Timeline representing the groups and experiment layout for injury, transplant, and drug administration. (**B**) BMS score of Control and PAB groups. (**C**) BMS score of transplant groups NaChBac and NaChBac + PAB. N = 6. Data represented as mean ± SD. The 2-way ANOVA test analysis did not reveal significant differences among groups.

Next, we studied the effect of local administration of the non-toxic analogue of blebbistatin, para-amino blebbistatin (PAB), immediately after injury alone or in combination with acute transplantation of NaChBac expressing DRGs. For this, we performed a double hemisection in animals at T7–T10 levels, and the drug or only media (as Control) were injected alone or together with the transplant (Figure 4A). Locomotor evaluation using the Basso Mouse Scale (BMS) showed that PAB administration had no effect compared to control animals (Figure 4B). When administered together with a NaChBac DRGs transplant, the effect of PAB, although not significant, seems to be detrimental (Figure 4C). These data illustrate the need for further additional in vitro and in vivo evaluations for the successful combinatory strategies translated from specific in vitro assays to the in vivo approaches.

## 3. Discussion

Epothilone B and blebbistatin, both together and individually, have been shown to increase axonal regeneration and induce recovery after CNS damage [14,17,18,19]. Thus, we hypothesized that the combination of these cytoskeleton-modulating drugs, which act upon different components of the growth cone, microtubules, and actin, respectively, should have a synergistic effect and improve axonal length significantly. When plated over a Non-Permissive substrate such as CSPGs simulating an injury environment, we observed that blebbistatin or epothilone B individually significantly increased axonal length of DRG neurons, but the combination reverted this effect to significantly shorter axonal lengths. When we studied the growth cone structure of DRG neurons plated over Non-Permissive substrate treated with the combination of both drugs, we found an increase in the growth cone area, similar to that found in the non-treated group. This structure was reminiscent of the “splayed” morphology found in DRG growth cones in the presence of aggrecans, which was reverted by reorganization in actin structure and de-bundling of microtubules by blebbistatin, effectively overcoming the aggrecan “splayed” morphology [16]. Indeed, we found that blebbistatin reverted this “splayed” morphology to a functional state.

Axonal extension is regulated by the cytoskeletal organization of the growth cone, which consists of a peripheral (P) domain rich in actin filaments, a transition (T) domain containing actomyosin arcs, and a central (C) domain dominated by microtubules [20]. Actin polymerization in the P domain drives protrusion, whereas actomyosin contractility in the T domain generates retrograde forces that restrict growth; inhibition of non-muscle myosin II with blebbistatin alleviates this restraint and promotes elongation [21,22]. Microtubule dynamics in the C domain, controlled by polymerization and catastrophe, are equally critical, and stabilizing agents such as epothilone B can enhance extension [23]. However, the interplay between actin and microtubules requires dynamic balance. Stabilizing microtubules while simultaneously reducing actomyosin tension may prevent proper cytoskeletal remodeling. While blebbistatin alone reduces retrograde actin tension, microtubules de-bundle to support elongation with functional regeneration of the injured peripheral nerve [19]. We postulate that the combination with epothilone B possibly inhibits the de-bundling of the microtubules, nullifying the positive effect of blebbistatin alone; therefore, multiple modifications of the cytoskeleton need to be well encompassed for a synergistic effect; otherwise, as we demonstrated, it would result in disrupting coordinated growth cone progression.

Since NaChBac expression does not increase neurite length in DRGs by itself, but when combined with blebbistatin treatment, it produces a significant increase under Non-permissive coatings, we tested in vivo the combinatory treatment of the DRG, modified to express NaChBac with the local administration of para-amino blebbistatin, a stable analog [24]. However, unexpectedly, none of the para-amino-blebbistatin-treated groups gave any locomotor beneficial effect, and, more surprisingly, the combination with NaChBac was detrimental in comparison with the transplant alone. Recent studies highlighted the importance of RhoA, which restricts microtubule extension [22]. Neurons isolated from knockout RhoA mice result in axonal elongation in vitro [16,25]. Nevertheless, one of these studies showed that the RhoA knockout does not improve axon extension after SCI, but rather inhibits it. This is because the RhoA pathway plays an important role in limiting astrogliosis, which increases in a RhoA knockout model, thereby increasing astrocytic reactivity, consequently preventing axonal regeneration. However, selective ablation of RhoA only in neurons led to a significant increase in axonal regeneration after injury, suggesting that the role of RhoA is a dual one [25]. Therefore, we hypothesized a similar effect in the downstream RhoA effector, NMMII, and its inhibitor, blebbistatin. The application of blebbistatin in our experimental approach is neither selective nor continuous. Hence, we theorize that the immediate effect of blebbistatin was to increase astrocytic reactivity, preventing other mechanisms of action for improving functional locomotion. These studies highlight the effects of some treatments over other cell types and how they can provide an additional benefit or restrict regeneration, also highlighting the need for a time-dependent administration of treatments.

## 4. Conclusions and Future Perspectives

Our findings highlight several complexities surrounding CNS repair studies. The first, the several intricacies of cytoskeletal regulation in axonal regeneration. While both blebbistatin and epothilone B individually promoted axonal extension in vitro, their combination unexpectedly negated these benefits, underscoring the need for balanced actin–microtubule dynamics within the growth cone.

Second, we show in our in vivo experiments revealed that blebbistatin treatment, either alone or combined with NaChBac, did not improve functional outcomes and could even be detrimental. This discrepancy between in vitro and in vivo assays illustrates the limitations of simplified culture systems, where neuron-intrinsic effects do not recapitulate the influence of the surrounding tissue environment. Importantly, interactions with other cell types, such as astrocytes, likely modulate treatment outcomes and may counteract the pro-regenerative effects observed in isolated neurons.

Despite these challenges, the modulation of cytoskeletal dynamics remains a promising therapeutic avenue for CNS injury. Our study highlights the requirement of careful consideration of the interplay between actin and microtubules, as well as their interactions with non-neuronal cells. Together, these results suggest that successful strategies for CNS repair must integrate neuronal cytoskeletal remodeling with broader regulation of the injury milieu to bridge the gap between cellular assays and functional recovery. This includes selective or temporally controlled delivery, or by combining cytoskeletal modulators with strategies that limit glial scarring.

## 5. Materials and Methods

### 5.1. Dorsal Root Ganglia Isolation and Culture

Dorsal root ganglia (DRG) were isolated from decapitated neonate rats at P3–P4, as previously described [26]. Briefly, DRGs from 4–5 pups were dissected from all medullary segments, pooled, and collected in cold HBBS (#24020091, Gibco, Thermo Fisher Scientific, Waltham, MA, USA). After removal, residual spinal roots and nerves attached to the DRG were carefully cut under a stereoscope, and DRGs were collected and allowed to settle to remove HBBS solution. Then, they were digested in Collagenase IA (#C9891, Sigma, St. Louis, MO, USA) for 35 min. Collagenase solution was removed by allowing DRGs to settle, followed by 7 min digestion with TrypLE (#12563-011, Gibco, Thermo Fisher Scientific, Waltham, MA, USA). TrypLE solution was carefully discarded and digested DRGs were suspended in complete medium (MM: Dulbecco’s Modified Eagle Medium (DMEM) F12 (+ 10% fetal bovine serum (FBS) + 1% P/S (penicillin/streptomycin) (#15140122, Gibco, Thermo Fisher Scientific, Waltham, MA, USA) and 25 ng/µL NGF (#13257-019, Gibco, Thermo Fisher Scientific, Waltham, MA, USA), and mechanically dissociated with a 1000 µL blue pipette tip and seeded plated as described below.

### 5.2. Coatings

Firstly, 12 coverslips mm were placed in P24 cell culture plates and coated with poly-L-lysine (#P2636, Sigma, St. Louis, MO, USA) at a concentration of 20 µg/mL overnight at 37 °C, washed with ddH_2_O.

In short, after washing of poly-L-lysine coating with ddH_2_O; coating laminin plus CSPGs coating was prepared for the non-permissive condition, whereas laminin plus bovine serum albumin (BSA) coating was prepared for the permissive condition. For permissive substrate, a coating solution of 10 µg/mL of laminin and 2 µg/mL BSA (#10735078001, Roche, Basel, Switzerland) was prepared in 1x phosphate-buffered saline for permissive substrate and added to the coverslips for 3 h at 37 °C.

Similarly, for the non-permissive substrate, a coating solution of 10 µg/mL of laminin and 2 µg/mL CSPGs (#CC117, Sigma, St. Louis, MO, USA) was prepared in 1x phosphate-buffered saline for the non-permissive substrate and added to the coverslips for 3 h at 37 °C.

### 5.3. Neurite Assay Experiments

In order to successfully analyze the neurite length of the DRG neurons, we used low-density cultures. DRGs were seeded at a concentration of 10,000 cells/well, in 500 mL of complete MM and NGF over chondroitin sulphate proteoglycans (CSPGs, non-permissive) or bovine serum albumin (BSA, permissive) substrates. Four hours after plating, the drug of choice (blebbistatin, epothilone B, or a combination of both) was added. The following concentrations were used: epothilone B (Epo B) at 0.1 nM; blebbistatin (Blebb) at 15 µM; and the combinations of both. Cells were fixed 20 h after drug application using 4% PFA and 4% sucrose for 20 min and washed with PBS three times.

Following this, immunocytochemistry for the visualization of β-III-tubulin and actin was performed. Cells were first permeabilized and blocked in Blocking Solution: 0.5% Triton-TX100 + 3% normal goat serum (NGS) for an hour at RT. Primary antibody for β-III-tubulin used was mouse Anti-alpha-Tubulin Purified (1:400, #11-250-C100, Exbio, Vestec, Czechia), and was added in blocking solution overnight at 4 °C. Actin (1:400, #ab176756, Abcam, Cambridge, UK) was added with a secondary antibody, AlexaFluor 488 anti-mouse, for an hour at RT, and then washed. Coverslips were counterstained with 4′,6-diamidino-2-phenylindole DAPI (1:1000, #D9542, Sigma, St. Louis, MO, USA) and mounted with Mowiol^®^ (#81381, Sigma, St. Louis, MO, USA).

### 5.4. Image Analysis

Images were obtained in 20× magnification using Zeiss Aperio Apotome microscope. Total neurite length was defined as the total length of all dendrites and was measured using NeuronJ plugin in ImageJ 1.x software in microns using the adequate scale. Briefly, images were opened and converted to 8 bits. The same threshold was applied to all images. Images were then opened in NeuronJ, and dendrites were manually traced. Total dendrite length per neuron in microns was expressed per neuron, replicates = 4.

Growth cone images were obtained by imaging at 100× using Zeiss Aperio Apotome microscope. Growth cone area was defined as the actin-positive area at the microtubule end of the growth cone. This was manually traced and expressed as area in µm^2^.

### 5.5. Spinal Cord Injury

This model consists of a double lateral, opposite hemisection model that ensures a complete injury but maintains a relay zone between two lateral hemisections. This was achieved by a T7–T10 double unilateral complete hemisection model as described in [27]. Briefly, a T7–T10 laminectomy was performed to expose the spinal cord, and an incision was made using a micro-scalpel blade (#72-2201 Sharpoint Angiotech, Reading, PA, USA) on the right T7 side until the midline. This incision was performed twice to ensure complete severing of the spinal cord. Similarly, an incision was made from the left T10 level to the midline. The muscle and skin layers were then separately sutured using 4/0 monosyn suture (#G2022004, Braun, Kronberg im Taunus, Germany), followed by a drop of Histoacryl to prevent the opening of the wound.

Animals were distributed into 4 groups: (1) SCI and 3 μL media (Control); (2) SCI and one administration of 15 μM para-amino blebbistatin in 3 μL (PAB); (3) SCI and transplant of 1 × 10^6^ dissociated DRGs cells expressing NaChBac in 3 μL; (4) SCI, transplant of 1 × 10^6^ dissociated DRGs cells expressing NaChBac with one administration of 15 μM PAB in 3 μL (*n* = 7–8 animals/group).

Following surgery, mice were kept warm using heating pads, and food was kept on the cage floor. All animals were subcutaneously administered buprenorphine twice a day (0.1 mg/kg) for 4 days after surgery and enrofloxine once a day for a week after surgery (5 mg/kg). Buprenorphine is an analgesic administered to mice after SCI to provide postoperative pain relief to mice. Enroflaxine is a wide-range antibiotic that is used to prevent urinary tract infections and wound infections. Manual bladder drainage was performed twice or once a day until recovery (if any) or experimental endpoint. Immunosuppressant cyclosporine (once a day, 20 mg/kg) was administered from one day before transplant until the experimental endpoint. Animals were carefully checked for endpoint symptoms.

### 5.6. Functional Evaluation

Motor function was evaluated by using the Basso Mouse Scale (BMS) [28], an established scale that evaluates locomotor function based on the specific guidelines described by the scale. This scale evaluates ankle and paw movement, trunk stability, and step coordination of mice. For this test, animals were placed in an open field twice a week and recorded for 3 min only. Videos were analyzed by a trained, blinded investigator, and a maximum score was given for each paw according to the scale. Data was then represented as the score of the mice over time.

### 5.7. Statistical Analysis

All statistical analysis was performed using GraphPad Prism 10 Software. Shapiro–Wilk normality test was performed to ensure data set normality (Gaussian distribution). Comparisons between the two groups were carried out using the two-tailed *t*-test. ANOVA was performed to compare more than two groups with single variables, followed by Tukey’s post test for typical data sets. The confidence level for all tests was set at 95%. All bar plots are represented as mean ± SD. P values are represented as: * *p* < 0.05; ** *p* < 0.01; *** *p* < 0.001; and **** *p* < 0.0001.

## Data Availability

The raw data required to reproduce these findings will be available at https://doi.org/10.6084/m9.figshare.30406312 or by author’s request.
